# Acute Intrathoracic Tuberculosis in Children and Adolescents with Community-Acquired Pneumonia in an Area with an Intermediate Disease Burden

**DOI:** 10.3390/pediatric14010011

**Published:** 2022-02-05

**Authors:** Claudia Roya-Pabón, Andrea Restrepo, Olga Morales, Catalina Arango, María Angélica Maya, Marcela Bermúdez, Lucelly López, Carlos Garcés, Mónica Trujillo, Luisa Fernanda Carmona, Margarita Rosa Giraldo, Lázaro A. Vélez, Zulma Vanessa Rueda

**Affiliations:** 1 Grupo Pediaciencias, Departamento de Pediatría y Puericultura, Universidad de Antioquia UdeA, Medellin 050010, Colombia; clalili@gmail.com (C.R.-P.); olmmunera@yahoo.com (O.M.); catarango52@hotmail.com (C.A.); calichegarces@hotmail.com (C.G.); 2 Departamento de Pediatría, Hospital Universitario San Vicente Fundación, Medellin 050010, Colombia; 3 Pima County Health Department, Tuberculosis Clinic, Tucson, AZ 85713, USA; 4 Departamento de Pediatría, Hospital Pablo Tobón Uribe, Medellin 050010, Colombia; avrestrepo33@gmail.com (A.R.); trupitv@gmail.com (M.T.); 5 Departamento de Pediatría, Universidad CES, Medellin 050010, Colombia; 6 Unidad de Enfermedades Infecciosas, Hospital Universitario San Vicente Fundación, Medellin 050010, Colombia; mangelicamaya@gmail.com (M.A.M.); lazarovelezg@gmail.com (L.A.V.); 7 Grupo Investigador de Problemas en Enfermedades Infecciosas (GRIPE), Facultad de Medicina, Universidad de Antioquia UdeA, Medellin 050010, Colombia; marcelita8510@gmail.com; 8 Grupo de Investigación en Salud Pública, Facultad de Medicina, Universidad Pontificia Bolivariana, Medellin 050031, Colombia; lucellyl@gmail.com; 9 Section of Pediatric Infectious Diseases, Clínica Universitaria Bolivariana, Medellin 050010, Colombia; 10 Secretaría Seccional de Salud y Protección Social de Antioquia, Gobernación de Antioquia, Medellin 050010, Colombia; lufe2346@hotmail.com (L.F.C.); giralrosita@gmail.com (M.R.G.); 11 Department of Medical Microbiology and Infectious Diseases, University of Manitoba, Winnipeg, MB R3E 0J9, Canada

**Keywords:** cohort study, children, adolescents, community-acquired pneumonia, intrathoracic tuberculosis, acute tuberculosis

## Abstract

Tuberculosis (TB) in the pediatric population is a major challenge. Our objective was to describe the clinical and microbiological characteristics, radiological patterns, and treatment outcomes of children and adolescents (from 1 month to 17 years) with community-acquired pneumonia (CAP) caused by TB. We performed a prospective cohort study of a pediatric population between 1 month and 17 years of age and hospitalized in Medellín, Colombia, with the diagnosis of radiologically confirmed CAP that had ≤ 15 days of symptoms. The mycobacterial culture of induced sputum was used for the bacteriological confirmation; the history of TB contact, a tuberculin skin test, and clinical improvement with treatment were used to identify microbiologically negative TB cases. Among 499 children with CAP, TB was diagnosed in 12 (2.4%), of which 10 had less than 8 days of a cough, 10 had alveolar opacities, 9 were younger than 5 years old, and 2 had close contact with a TB patient. Among the TB cases, 50% (6) had microbiological confirmation, 8 had viral and/or bacterial confirmation, one patient had multidrug-resistant TB, and 10/12 had non-severe pneumonia. In countries with an intermediate TB burden, *Mycobacterium tuberculosis* should be included in the etiological differential diagnosis (as a cause or coinfection) of both pneumonia and severe CAP in the pediatric population.

## 1. Introduction

A tuberculosis (TB) diagnosis in children is a major challenge due to the difficulty of obtaining appropriate samples, the low microbiological yield, and the limitation of the clinical definitions that require more than 2 weeks of signs/symptoms to be suggestive of tuberculosis (persistent >2 weeks, unremitting cough, weight loss/failure to thrive, persistent unexplained fever, persistent unexplained lethargy, or decrease in playfulness/activity reported by the parent/caregiver) [1]. However, this clinical definition may be a late approach to diagnosing TB.

When pulmonary TB presents as an acute illness, it is indistinguishable from community-acquired pneumonia (CAP) caused by other pathogens [2]. This often leads to a delay in diagnosis with increased TB morbidity and mortality [3].

Colombia is a tuberculosis endemic country with an intermediate burden (20.88/100,000 cases per population/year). In 2020, a total of 11,390 cases were reported, with an incidence in children under 15 years of 2.7%, and of these, 39.3% corresponding to children under 5 years of age [4].

CAP continues to be the leading cause of mortality in children under 5 years of age in low- and middle-income countries [5]. The most recently published studies have revealed that, in these children, CAP is caused by viruses (61–66%), bacteria (27%), atypical bacteria (7.2–20%), and *Mycobacterium tuberculosis (M. tb)* (2.9–5.9%) [6,7,8,9,10]. Furthermore, the coinfection of viruses and bacteria is not infrequent [6,7,8,11].

Like CAP, TB is also an important cause of illness and death worldwide, despite being a preventable and curable disease. In 2020, there were a total of 9.9 million TB disease cases worldwide, with 1,089,000 occurring in children, showing a mortality of 16% [12]. A systematic review of patients with severe pneumonia in children under 5 years of age in countries with a high burden of TB found 7.5% with confirmed cases of acute-onset TB [13]. Most of those studies were conducted in countries with a high HIV prevalence. Oliwa et al. [13] highlight that “previous studies of pneumonia in infants and young children might have underestimated the contribution of tuberculosis as a direct cause or comorbidity of acute community-acquired pneumonia in children because of the difficulties of microbiological confirmation in this age group in resource-restricted tuberculosis endemic settings”.

Our goal was to describe the clinical characteristics, radiological patterns, microbiological features, and treatment outcomes in the pediatric population of between 1 month and 17 years old with CAP caused by *M.tb* in an intermediate TB burden country with a low HIV prevalence.

## 2. Materials and Methods

Study type: Prospective cohort study.

Study population: We included a pediatric population of between 1 month and 17 years of age with a diagnosis of community-acquired pneumonia that required hospitalization in 13 institutions of medium- and high-complexity care from Medellín, Bello, Envigado, Itaguí, Colombia. 

Inclusion criteria: Children and adolescents with a diagnosis of CAP and with respiratory symptoms ≤15 days duration. CAP was defined as the presence of lung opacities (alveolar or interstitial) on a chest X-ray and one of the following symptoms or signs: axillary fever ≥38.3 °C; tachypnea according to the age of the patient; the finding of rhonchus and/or rales and/or wheezing on auscultation. 

Exclusion criteria: Children and adolescents hospitalized in the last 15 days, patients with previous antibiotic treatment for more than 72 h at the time of hospital admission, patients with primary or severe acquired immunodeficiency, cystic fibrosis, bronchiolitis obliterans, neurological (including cerebral palsy) or neuromuscular disorders, psychiatric disorders that did not allow the patient to assent, congenital metabolic disorders, bronchiolitis (first sibilant episode in children less than 2 years), hematological malignancies, neutropenia (<500 cells/mm^3^), primary ciliary dyskinesia, acute immunodeficiency syndrome (AIDS) or HIV infection with a CD4 count of less than 15% in children of 5 years or younger, or a CD4 cell count <200 cells/mm^3^ in children older than 5 years, treatment with prednisolone ≥1 mg/kg/day or its equivalent for more than 8 days, or other immunosuppressive drugs such as cyclosporine, methotrexate, mycophenolate mofetil, cyclophosphamide, azathioprine, and fluorouracil.

Patient enrollment: Between August 2011 and September 2012, children and adolescents with CAP admitted through the pediatric emergency room and requiring hospitalization in 13 participating institutions. 

Ethics approval: This study was approved by the Ethics Committee of the Medical School of the Universidad de Antioquia and by each participating institution. All parents or legal guardians and the child were asked to voluntarily participate in the study, and after explaining its purpose, informed consent was signed by the parents (legal guardians) and informed assent was obtained in children >7 years old. All patients had the same access to health care and received treatment according to the standard of care.

Procedures and laboratory tests: Patient enrollment was carried out by general practitioners trained by the study investigators following standardized procedures. Children and adolescents who met the inclusion criteria and did not meet any exclusion criteria were enrolled. The data collected for each patient included sociodemographic, clinical, and laboratory information, history of TB exposure, past medical history, previous immunizations or treatments; these were filled out in a data collection form designed by the investigators. 

Severe pneumonia was classified according to the World Health Organization (WHO) classification of childhood pneumonia, i.e., non-severe (pneumonia): fast breathing; severe: chest indrawing; very severe: not able to drink, persistent vomiting, convulsions, lethargic or unconscious, stridor in a calm child, or severe malnutrition.

A trained nurse administered the tuberculin skin test (Tuberculin PPD RT-23, SSI 2 T.U/0.1 mL, Statens Serum Institut^®^, Copenhagen, Denmark) to all accepted children and adolescents, according to CDC guidelines. The reading was performed 48 to 72 h later and measured in millimeters of induration. 

A chest X-ray was obtained for each patient, and readings were performed by the radiologists of each institution.

A blood sample (for aerobic bacterial culture and serology for atypical microorganisms), a urine sample (for *Streptococcus pneumoniae* and *Legionella pneumophila* antigens), and 3 induced sputum (IS) samples (for viruses, pyogenic bacteria, and *M.tb)* were collected from each patient. 

IS was obtained after inhalation of β-2 agonist (200 µg) and nebulization of 5% hypertonic saline. IS samples were collected by nasopharyngeal aspirate (children younger than 5 years) or by generating a cough through forced expiration and assisted coughing. Sputum induction was a procedure that was performed in all ages. Continuous monitoring before, during, and one hour after the procedure was performed, and no complications were recorded. 

Additionally, a nasopharyngeal swab was collected for viral detection with direct and indirect immunofluorescence for *Bordetella pertussis*. 

One of the IS samples was stained with auramine–rhodamine and inoculated in solid and liquid cultures for *M. tb* (Löwenstein–Jensen, thin-layer agar, and BD Bactec ™ MGIT ™ 960, Sparks, MD, USA). The samples were transported at 2–8 °C to the laboratory. The IS and NS samples were stored at −80 °C until processing.

In addition to the case definition of CAP, the following definitions were applied for intrathoracic TB. Microbiologically confirmed TB was defined as a positive culture for *M. Tb*. TB without microbiological confirmation: culture negative for *M.tb* and history of household or close contact with a confirmed TB case or immunological evidence of previous exposure to *M.tb* (positive PPD ≥10 mm) and an adequate clinical response to the TB treatment.

The follow-up on the children and adolescents with CAP included in the study was conducted by pediatric pulmonologists.

One month after discharge from the hospital, an additional evaluation was performed by a pediatric infectious disease specialist in those patients with suspected or confirmed TB as well as those with a diagnosis of latent tuberculosis infection to rule out TB. 

In addition, a follow-up phone call was made by the medical and nursing team hired by this research project at 3, 6, and 12 months after recruitment.

Statistical analysis: The data were included in a database designed in Microsoft Access^®^ 2007, version 12.0, and then exported to an SPSS^®^ version 22 for analysis. To summarize the variables, absolute and percentage frequencies were used.

## 3. Results

**Figure 1:**
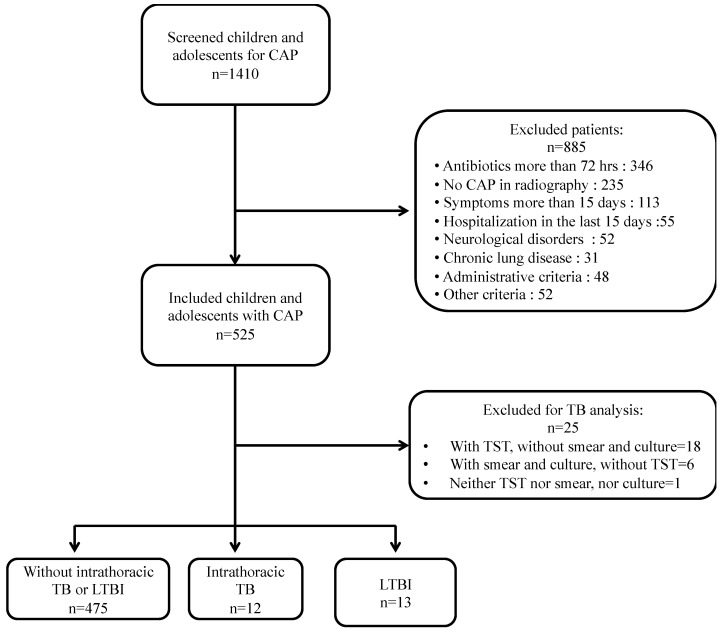
Flow chart of included patients.

The study evaluated 1410 children and adolescents, and 525 met the inclusion criteria. Among them, 26 patients were excluded because they did not have all the TB tests (Figure 1).

Among the 499 children and adolescents with CAP and complete TB tests, 12 were diagnosed with intrathoracic TB and 13 with a latent TB infection (LTBI). Those with intrathoracic TB were referred by the study investigators to their pediatricians and the local TB control program to start treatment and to perform a TB contact tracing investigation. All patients in the study had received BCG at birth, and none of them had HIV.

In patients with intrathoracic TB, the median age was 3 years, and 75% (9/12) were younger than 5 years. Most patients with intrathoracic TB had less than 8 days of a cough (83.3%, 10/12), 16.7% (2/12) had a history of being a close contact with a TB case, one had diabetes mellitus (DM) (8.3%), and one had malnutrition (8.3%). In addition, 83.3% (10/12) of the TB cases had non-severe pneumonia, 33.3% (4/12) had a history of previous CAP episodes before the current one, 83.3% (10/12) had low socio-economic status, and 16.7% had oxygen saturation below 90% (Table 1). 

The diagnosis of intrathoracic TB was made by a bacteriological confirmation in IS culture for 50% (6/12) of cases and one patient had multidrug-resistant TB (MDR-TB). Alveolar opacities were the most frequent radiological abnormalities (83.3%). Of the 12 patients with intrathoracic TB, eight had coinfections and six had more than two pathogens (Table 2). TB treatment was well tolerated by all children and adolescents. During the follow-up, 83.3% (10/12) finished TB treatment and showed clinical improvement. Two children were lost to follow-up. None of the patients died (Table 2).

Of the 13 patients with LTBI, 11 were eligible to receive latent tuberculosis infection (LTBI) treatment but only five of them received it. Among the reasons for not receiving LTBI treatment were: four patients’ mothers did not accept LTBI treatment for their children, two because their primary physician disagreed with the treatment, one had an administrative barrier, and one was lost at follow-up. All patients tolerated the treatment well, and none developed intrathoracic TB during the follow-up.

## 4. Discussion

Despite the fact that Colombia has an intermediate TB burden, an incidence of pediatric CAP due to TB has not been reported. In our study, the incidence of TB in patients with CAP was 2.4%. In other reports by authors in countries with a high burden of TB, the incidence varies between 2.9% and 5.9% [6,10]. An incidence as high as 18.9% has been reported in patients with severe CAP in Uganda [3]. This resulted in a recommendation to rule out TB in every patient with severe CAP in that country. The above finding can be explained by the fact that Uganda is a country with a high TB burden (200 cases/100,000 population) and with a high prevalence of HIV infection [3,12]. In our study, none of the children had an HIV infection when TB was diagnosed, and 83.3% (10/12) of the TB cases had non-severe CAP. Therefore, *M.tb* should be considered as a CAP pathogen even in non-severe cases. 

We found no clinical or radiological differences between children and adolescents with CAP with TB and patients with CAP caused by other pathogens, similar to what was reported in a systematic review of cases from endemic countries with a high burden of TB [13]. 

Although in our study only two cases of intrathoracic TB had a history of known close contact with confirmed TB, it is extremely important to obtain a history of exposure to people with TB in children and adolescents presenting with CAP. TB exposure is a high-risk factor for developing a TB disease, as demonstrated by Martinez et al. [14], who found that exposed children had a 2-year cumulative risk of developing TB disease from 5.2% to 7.6%, and Nantongo et al. [3,14], who described that children with a history of exposure to a smear-positive TB patient in the past year were three times more likely to develop TB [2,13].

There are well-known risk factors strongly related to the progression from infection to TB disease, such as young patients <5 years of age caused by an immature immune system and comorbidities such as DM and malnutrition present in our study, which in turn predispose patients to an acute form of the disease [15]. In our study, children younger than 5 years represented the majority of intrathoracic TB cases (75%), and two cases with severe disease belonged to this age group. Thus, early diagnosis and treatment of TB is important, especially in young children [16] who are at greater risk of a severe disease and more complications.

Our study did not find any symptoms/signs suggestive of TB [1] in patients with CAP and TB. With our findings, we also want to open a debate into whether it is time to change the clinical definitions of tuberculosis in children and adolescents, as it has been reported in adults that up to 77.6% of people with TB are asymptomatic [17]. Cavallazzi et al. [18] reported 60 (0.86%) patients with TB in a study of 6976 adults with CAP. The authors describe five clinical findings that could predict TB in patients presented with CAP: night sweats, hemoptysis, weight loss, previous exposure to TB, and pulmonary opacities in the upper lobes. Nonetheless, the first three clinical findings do not usually apply to a pediatric population because children with TB do not always have these symptoms, and the most common history is of no weight gain, failure to thrive, and decreased level of activity [16].

Microbiological confirmation of intrathoracic TB was achieved in 1.2% (6/499) of cases with CAP by collecting adequate samples of IS. Other reports describe a 5–8% microbiological confirmation with IS and/or gastric aspirates, this being as high as 15% in places with a high burden of TB [6,19]. In addition, we had one case with MDR-TB, which alerted us to the presence of these cases in our population and highlighted the importance of having ongoing epidemiological surveillance and always pursuing microbiological confirmation in children despite the difficulties due to their paucibacillary nature and the challenge of collecting specimens in young patients. 

In the last few years, there has been more insight into the role of coinfections with *M.tb* to influence the immune response, whether predisposing the person to acquire the infection, in disease progression, or by determining the severity or the capacity to control the disease [13,20,21]. In our study one patient had severe CAP, intrathoracic TB, and viral coinfection. In a systematic review by Oliwa et al. [13], TB coinfection with other pathogens had more severe clinical presentations and worse outcomes, including mortality. In our study, we found viral and/or bacterial coinfections in 66.6% (8/12) of the cases and none of them had severe pneumonia and none died. In these cases, TB could be present as a subclinical disease [22] where the acute symptoms are due to the other microorganism or as a case of a clinical TB disease. 

A recent systematic review and individual participant meta-analysis continue to confirm that the risk of developing TB among exposed infants and young children is very high (19%). The majority (83%) of these children who developed a TB infection progressed to TB disease in the first 90 days [14]. In our study, 45% (5/11) of the patients with LTBI were younger than 5 years of age and did not receive any LTBI treatment. Therefore, we consider LTBI treatment as a key intervention to control TB from an early stage that must be administered without delay.

One limitation of our study was that we only collected one IS sample as we did not expect to find TB cases as a cause of acute CAP, and molecular tests were not available at the time when the study was conducted, which probably would have increased the cases of TB or confirmed those with a negative culture [23].

## 5. Conclusions

In countries with an intermediate TB burden, *M.tb* should be included in the etiological differential diagnosis (as a cause or coinfection) of both severe and non-severe CAP in children and adolescents. We invite researchers and clinicians around the world to debate whether the current clinical definitions of TB in children may be a late approach to diagnosing TB as it has been documented in adults that up to 77.6% of people with TB are asymptomatic [17]. It is important to conduct more research in children with CAP that includes a systematic search for *M.tb,* including molecular testing, in countries with an intermediate TB burden as well as develop screening tools that allow identifying incipient and sub-clinical TB [22] in children.

## Figures and Tables

**Table 1:** Baseline characteristics of children and adolescents with intrathoracic TB and community-acquired pneumonia.

Variables	Children and Adolescents with CAP and Intrathoracic TB N = 12 (%)	Children and Adolescents with CAP without Intrathoracic TB N = 487 (%)
Female	9 (75)	236 (48.5)
Children ≤ 5 years of age	9(75)	409 (84.0)
Children > 5 years of age	3(25)	78 (16)
Prior antibiotic use Last 3 months Last 48 h	1 (8.3) 2 (16.7)	115 (23.8) 34 (7.0)
Close TB contact case	2 (16.7)	17 (3.5)
Cough	12 (100)	476 (97.7)
Cough < 8 days	9 (75)	332 (68.2)
Fever	12 (100)	452 (92.8)
Irritability	7 (58.3)	222 (45.6)
Chest pain	5 (41.7)	120 (24.6)
Severity of pneumonia *		
Non-severe Severe Very severe	10 (83.3) 2 (16.7) 0 (0)	357 (73.3) 115 (23.6) 15 (3.1)
Radiological abnormalities Alveolar opacities Interstitial opacities Pleural effusion	10 (83.33) 5 (41.66) 1 (8.33)	352 (70.54)258 (51.70)24 (4.80)
Coinfections	8 (66.7)	172 (35.3)
Comorbidities Asthma Low birth weight Diabetes mellitus Malnutrition	1 (8.3) 2 (16.7) 1 (8.3) 1 (8.3)	135 (27.8) 56 (11.6) 1 (0.2) 52 (10.7)
History of CAP	4 (33.3)	129 (26.6)
Low socio-economic status	10 (83.3)	366 (78.5)
Oxygen saturation below 90%	2 (16.7)	165 (33.9)

CAP: community-acquired pneumonia; TB: tuberculosis; low socio-economic status: Colombia classified people according to their income in 6 categories, stratum 1 being the lowest income and 6 the highest income. This variable includes strata 1, 2, and 3. * Severity of pneumonia: non-severe (pneumonia): fast breathing; severe: chest indrawing; very severe: not able to drink, persistent vomiting, convulsions, lethargic or unconscious, stridor in a calm child, or severe malnutrition.

**Table 2:** Radiological, epidemiological, tuberculin skin test, and microbiological characteristics in 12 children and adolescents with community-acquired pneumonia and intrathoracic TB.

Case	Age	Close Contact with TB Case	TST (mm)	Radiological Abnormalities	Microbiological (Culture)	Coinfections during CAP Onset	Severity	Follow-Up
1	1 y.o.	Negative	Administered, but problems with TST reading	Lobar consolidation and diffuse interstitial opacities	Positive	Respiratory syncytial virus and influenza virus	Severe	Clinical improvement until last follow-up, lost in the second month after TB treatment started
2	1 y.o.	Negative	0	Focal interstitial opacity	Positive	Rhinovirus and *Coxiella burnetii*	Non-severe	Completed treatment Clinical improvement
3	1 y.o.	Negative	0	Lobar consolidation	Positive	*Streptococcus pneumoniae* and *Mycoplasma pneumoniae* and *Bordetella pertussis* and *Coronavirus*	Non-severe	MDR-TB, completed treatment Clinical improvement
4	2 y.o.	Positive	13	Focal interstitial opacity	Positive	Rhinovirus and *Moraxella catarrhalis*	Non-severe	Completed treatment Clinical improvement,
5	2 y.o.	Negative	0	Lobar consolidation	Positive	Parainfluenza and influenza virus	Non-severe	completed treatment Clinical improvement
6	14 y.o.	Negative	17	Lobar consolidation	Positive	*Moraxella catarrhalis*	Non-severe	Completed treatment Clinical improvement,
7	2 y.o.	Negative	16	Diffuse consolidation	Negative	No	Severe	completed treatment Clinical improvement,
8	3 y.o.	Negative	16	Lobar consolidation	Negative	No	Non-severe	completed treatment Clinical improvement,
9	4 y.o.	Negative	13	Lobar consolidation	Negative	*Streptococcus pneumoniae*	Non-severe	lost, no treatment completion data Clinical improvement until last follow-up,
10	4 y.o.	Positive	15	Lobar consolidation and focal interstitial opacity	Negative	No	Non-severe	completed treatment Clinical improvement
11	10 y.o.	Negative	20	Diffuse consolidation and diffuse interstitial opacities and pleural effusion	Negative	No	Non-severe	Completed treatment Clinical improvement
12	12 y.o.	Negative	17	Lobar consolidation	Negative	Influenza virus and *Mycoplasma pneumoniae*	Non-severe	Completed treatment Clinical improvement

CAP: community-acquired pneumonia; MDR-TB: multi-drug resistant; RSV: respiratory syncytial virus; TB: tuberculosis; TST: tuberculin skin test; y.o: years old.

## Data Availability

The data presented in this study are available on request from the corresponding author. The data are not publicly available due to at the time we conducted the study, we did not request to the parents written permission to share the data in publicly accessible repository.
